# Genome-wide analysis of expression quantitative trait loci (eQTLs) reveals the regulatory architecture of gene expression variation in the storage roots of sweet potato

**DOI:** 10.1038/s41438-020-0314-4

**Published:** 2020-06-01

**Authors:** Lei Zhang, Yicheng Yu, Tianye Shi, Meng Kou, Jian Sun, Tao Xu, Qiang Li, Shaoyuan Wu, Qinghe Cao, Wenqian Hou, Zongyun Li

**Affiliations:** 1 Jiangsu Key Laboratory of Phylogenomics and Comparative Genomics, School of Life Sciences, Jiangsu Normal University, Xuzhou, 221116 Jiangsu Province People’s Republic of China; 2 Xuzhou Academy of Agricultural Sciences/Sweet Potato Research Institute, CAAS, Xuzhou, 221121 Jiangsu Province People’s Republic of China

**Keywords:** Gene expression, Genome-wide association studies, Gene regulation, Natural variation in plants, Agricultural genetics

## Abstract

Dissecting the genetic regulation of gene expression is critical for understanding phenotypic variation and species evolution. However, our understanding of the transcriptional variability in sweet potato remains limited. Here, we analyzed two publicly available datasets to explore the landscape of transcriptomic variations and its genetic basis in the storage roots of sweet potato. The comprehensive analysis identified a total of 724,438 high-confidence single nucleotide polymorphisms (SNPs) and 26,026 expressed genes. Expression quantitative trait locus (eQTL) analysis revealed 4408 eQTLs regulating the expression of 3646 genes, including 2261 local eQTLs and 2147 distant eQTLs. Two distant eQTL hotspots were found with target genes significantly enriched in specific functional classifications. By combining the information from regulatory network analyses, eQTLs and association mapping, we found that *IbMYB1-2* acts as a master regulator and is the major gene responsible for the activation of anthocyanin biosynthesis in the storage roots of sweet potato. Our study provides the first insight into the genetic architecture of genome-wide expression variation in sweet potato and can be used to investigate the potential effects of genetic variants on key agronomic traits in sweet potato.

## Introduction

Sweet potato, *Ipomoea batatas* (L.) Lam., is ranked as the seventh most important crop worldwide for feed, food, and fuel^1^. Due to its strong adaptability, stable yield and high nutritional value, it is considered a food security crop in many countries. The storage roots of sweet potato, which are mainly harvested and consumed, provide a rich source of *β*-carotene, dietary fiber, flavonoids, starch, and other nutrients for people^2^. Unraveling the regulatory mechanisms of their biosynthesis and accumulation will be critical to improve the yield and nutritional value of sweet potato.

Sweet potato is a self-incompatible hexaploid with a high degree of heterozygosity (2*n* = 6*x* = 90) and has a large genome size of 1.6 Gb^3^. Advances in discovering the molecular mechanisms of important agronomic traits for this outcrossing crop are highly limited because of its complicated genetic and genomic characteristics. Due to the importance of sweet potato to humans and the development of sequencing technologies, more resources have been generated in recent years, such as the haplotype-resolved genome of *I. batatas*^4^, wild genomes of the ancestors *Ipomoea triloba* and *Ipomoea trifida*^5,6^, resequencing and transcriptome datasets for cultivated sweet potato^5,7^. Utilizing these genomic resources, many genes that are associated with storage root development and nutrient accumulation have been identified^5,7,8^. In addition, linkage or association analysis has also identified several loci or candidate genes that are responsible for storage root-related traits^6,9–11^. However, our insights into the processes and genetic regulatory networks in the storage roots of sweet potato remain limited.

Gene expression is an important molecular phenotype that associates genetic variation with phenotypic variation, and changes in gene expression are likely to play key roles in phenotypic variation and species evolution^12,13^. Under different environmental conditions and at different developmental stages, the expression of genes is coordinately regulated both spatially and temporally in an organism^14^. Dissecting the regulatory relationships of these genes at the genome-wide level is of great importance in understanding the biological processes of a species^13,15^. One potentially powerful approach for revealing gene regulatory relationships is expression quantitative trait locus (eQTL) mapping^16–18^. With the development of sequencing technologies, genome-wide eQTL mapping has been conducted to construct gene regulatory networks in many plants, such as Arabidopsis^19^, lettuce^20^, maize^13,21,22^, rice^15^, tomato^23^, and melon^24^. In addition, gene regulatory networks constructed by eQTL mapping combined with the results from linkage or association mapping may help us identify candidate causative genes underlying various biological traits, such as flowering time^15^, anthocyanin content^20^, and maize kernel oil concentration^22^.

In this study, we explored the sequence diversity and gene expression profiles in storage roots across different sweet potato accessions, using two datasets generated from 88^7^ and 16^5^ sweet potato accessions. Our purpose was to reveal the gene regulatory networks employed in mature storage roots of sweet potato. We detected a total of 724,438 high-confidence single nucleotide polymorphisms (SNPs) and 26,026 expressed genes. Genome-wide association studies (GWAS) identified 4408 eQTLs regulating the expression of 3646 genes, including 2261 local eQTLs and 2147 distant eQTLs. We identified two distant eQTL hotspots with target genes significantly overrepresented in specific functional classifications. As a striking example, we constructed a genetic regulatory network for flavonoid biosynthesis in the storage roots of sweet potato. By integrating information from regulatory network analyses, eQTL and association mapping, we found that *IbMYB1-2* is the major gene responsible for the activation of anthocyanin biosynthesis in the storage roots of sweet potato. Our study will be useful in investigating the potential effects of genetic variants on key agronomic traits in sweet potato.

## Results

### SNP genotyping

A total of 104 sweet potato accessions^5,7^ with anthocyanin variation in root flesh were collected for SNP genotyping (Supplementary Table S1). Next-generation sequencing (NGS) data for these accessions (transcriptome dataset of mature storage roots for 88 accessions^7^ and resequencing dataset for 16 accessions^5^) were downloaded from National Genomics Data Center (NGDC)^25^ and National Center for Biotechnology Information (NCBI)^26^ databases, respectively. Due to the presence of redundancy, incompleteness and numerous misassemblies in the *I. batatas* cv. Taizhong6 haplotype genome assembly^5^, the diploid progenitor *I. trifida* was used as the reference genome. For each accession, the filtered fastq data were aligned to a modified version of the *I. trifida* genome (Supplementary Note).

Using diploid *I. trifida* as the reference, homologous SNPs (polymorphisms that occur within the same subgenome) were difficult to accurately identify due to the presence of homeologous SNPs (polymorphisms that represent differences among subgenomes). To filter out homeologous SNPs, we used a modified SWEEP algorithm by selecting only SNPs that were homozygous for one allele (reference or alternative allele) in some accessions and heterozygous in the remaining accessions^27^. Finally, 724,438 high-confidence SNPs with a missing rate ≤40% were identified using a series of filtering approaches described in the “Methods” section (Supplementary Fig. S2).

The genomic distribution and functional effects of these SNPs were further investigated using SnpEff^28^. A total of 513,808 SNPs (70.93%) were mapped to the coding sequences (CDSs) of 20,716 genes (high-confidence gene model set, version 3) (Supplementary Fig. S3), including 317,189 synonymous and 196,619 nonsynonymous SNPs. Among them, 2473 large-effect SNPs were found to have a potentially disabling impact on the function of 2151 genes due to premature stop codons, modified start or stop codons, induced disruptive splice variants, *etc*. Some of these greatly affected genes have been well studied in model plants, such as *Phytochrome A* (*PHYA*, itf15g14890)^29^ and *ABRE binding protein 1* (*AREB1*, itf06g22880)^30^ (Supplementary Table S3). These genes may be important for sweet potato growth and warrant further investigation. Protein domain analysis of these genes revealed that the NB-ARC domain (PF00931) and TIR domain (PF01582) were significantly overrepresented (*P* < 0.05) (Supplementary Table S4), which was consistent with findings in many plants^20,31,32^.

### Gene expression variability in the transcriptome dataset

We further analyzed the transcriptome data of 88 accessions to explore the gene expression profiles in mature storage roots. TPM (transcripts per million)^33^ values were calculated to quantify gene expression. A total of 26,026 genes were considered to be expressed in the transcriptome dataset, accounting for 80.63% of the annotated genes. Of these genes, 79.63% had a mean TPM value ≥ 1, 45.00% had a mean TPM value ≥ 10, and 4.60% had a mean TPM value ≥ 100 across all accessions (Supplementary Fig. S4). The top 100 highly expressed genes in mature storage roots were listed by the ordered of average TPM values (Supplementary Table S5). Protein family analysis of these genes revealed that the potato inhibitor I and DRM/ARP family were significantly enriched (*P* < 0.05) (Supplementary Table S6), which were also reported to be highly expressed in mature organs of other plants^34,35^, suggesting the conserved function of these genes in plants.

Gene expression was highly variable in mature storage roots among the 88 sweet potato accessions. First, a total of 18,681 genes (71.78% of all expressed genes) that were expressed in all accessions were defined as core expressed genes, whereas the remaining 7345 genes were detected in only some of the accessions and were defined as dispensable expressed genes (Supplementary Fig. S5). Second, the coefficient of variation (CV) for all genes was calculated, ranging from 0.14 to 7.92. A total of 6071 genes with CV values greater than 0.85 (3rd quartile value) were defined as highly variable genes. Gene ontology (GO) enrichment analysis of highly variable genes revealed that secondary metabolite and hormone metabolic processes were overrepresented, such as flavonol biosynthetic and auxin biosynthetic processes (Supplementary Fig. S6), which suggested that the levels of metabolites and hormones may vary greatly among different sweet potato accessions.

### Global eQTL mapping and analysis

**Fig. 1:**
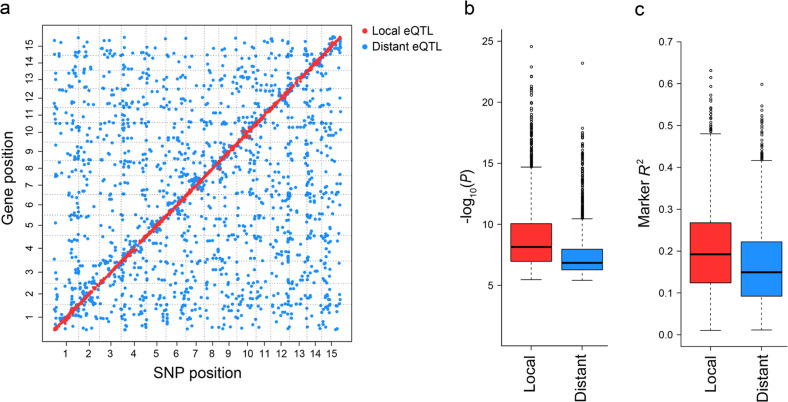
Global eQTL mapping. **a** eQTLs identified in the storage roots of sweet potato. The genomic positions of 4408 eQTLs (*x*-axis) for 3646 target genes (*y*-axis) in the genome are shown. **b** Comparison of the −log_10_(*P*) values of local and distant eQTLs. **c** Comparison of the effects of local and distant eQTLs

Genome-wide mapping of eQTLs could discover genetic regulatory networks that are active in the storage roots of sweet potato. After the removal of two outlier samples, a total of 86 accessions were used for further analysis. Considering the population structure, genetic relatedness among accessions and hidden confounding factors of expression variation, EMMAX^36^ was used to test for association between the expression levels of 24,635 genes and 390,241 SNPs. eQTL mapping showed that 78,633 SNPs were significantly associated with the expression trait of at least one gene at the genome-wide suggestive threshold (−log_10_ (*P*) > 5.39, *α* = 1). After grouping linked SNPs that were significantly associated with the same gene, a total of 4408 eQTL blocks were identified for 3646 genes (Fig. 1a, Supplementary Table S7). Among these genes, most (3100, 85.02%) had only 1 eQTL, whereas 409 genes had 2 eQTLs and 137 had 3 or more eQTLs (Supplementary Fig. S7).

eQTLs were further classified as local eQTLs and distant eQTLs based on their genomic positions and the genomic positions of their target genes. A total of 2261 local eQTLs and 2147 distant eQTLs were identified for 2261 and 1722 genes, respectively. As expected, local eQTLs were shown as a diagonal line, whereas distant eQTLs were unevenly distributed in the genome (Fig. 1a). The explained expression variation and −log_10_ (*P*) values between local and distant eQTLs were further compared (Fig. 1b, c). Overall, 63.86% of the local eQTLs and 33.61% of the distant eQTLs had −log_10_ (*P*) values greater than 7.39 (*α* = 0.01). A total of 47.24% of the local eQTLs and 31.69% of the distant eQTLs explained ≥20% of the corresponding gene expression variation. Our results suggested that local eQTLs played a greater role in determining expression variation than did distant eQTLs.

### Distant eQTL hotspots

**Fig. 2:**
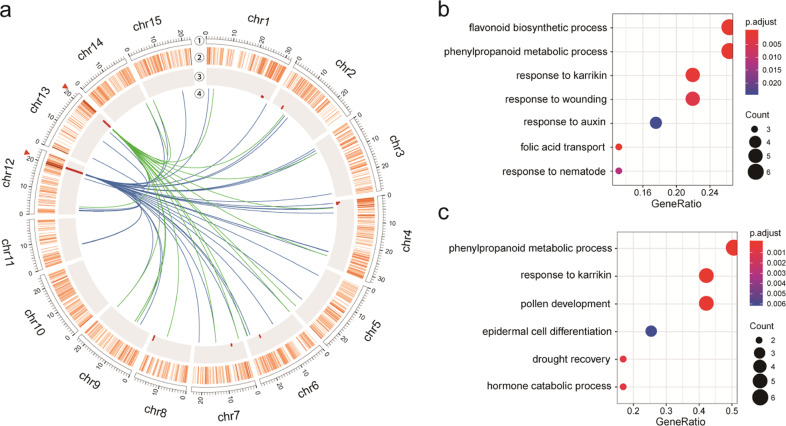
Distant eQTL hotspots in sweet potato. **a** Identification of distant eQTL hotspots. The two most significant distant eQTL hotspots are marked with red triangles on chromosomes 12 and 13. ①, fifteen chromosomes; ②, heatmap showing the number of target genes in a 200 Kb window along the chromosome; ③, histogram showing the −log_10_(*P*-value) for each eQTL hotspot; ④, the two most significant eQTL hotspots are displayed along their target genes in the genome. **b** GO (biological process) enrichment analysis of target genes regulated by eQTL hotspot on chromosome 12. **c** GO (biological process) enrichment analysis of target genes regulated by eQTL hotspot on chromosome 13

Distant eQTL hotspot may contain master regulators that can control the expression of many genes. By analyzing the distribution of distant eQTLs, a total of 10 distant eQTL hotspots (*P*_adjusted_ < 0.05) were identified (total size: 3 Mb, mean size: 355 Kb) across the whole genome, which regulated the expression of 158 target genes (Fig. 2a, Supplementary Table S8). GO enrichment analysis was further performed on the target genes for each distant eQTL hotspot, and only two distant eQTL hotspots were found to have significantly overrepresented biological processes (*P* < 0.05) (Fig. 2b, c). These two distant eQTL hotspots, one on chromosome 12 from 20.42 Mb to 20.96 Mb (*P*_adjusted_ = 6.61 × 10^−35^) and another on chromosome 13 from 21.32 Mb to 21.71 Mb (*P*_adjusted_ = 2.07 × 10^−11^), were the most significant distant eQTL hotspots (Fig. 2a).

In the eQTL hotspot on chromosome 12, significant distant eQTLs were identified for 47 genes that participate in multiple biological processes, such as flavonoid biosynthetic process, folic acid transport, and response to abiotic stimulus (Fig. 2b). By comparing the intervals of distant eQTLs for these 47 genes, a 540 Kb overlapping region was identified. According to the gene annotation of the *I. trifida* genome, there were 74 genes in the overlapping region. Interestingly, *IbMYB1-2*, encoding an R2R3-MYB transcription factor that is known to control anthocyanin biosynthesis^37,38^, is found in this region. In addition, a local eQTL for *IbMYB1-2* was detected. Coexpression analysis showed that 34 genes were strongly coexpressed with *IbMYB1-2* (*P* < 0.05). Thus, *IbMYB1-2* is a strong candidate master regulator in this eQTL hotspot. This finding contributes to a deeper understanding of the new regulatory roles of *IbMYB1-2*. In the eQTL hotspot on chromosome 13, significant distant eQTLs were identified for 20 genes that also participate in multiple biological processes, such as pollen development and phenylpropanoid metabolic process (Fig. 2c). By comparing the intervals of distant eQTLs for these 20 genes, a 390 Kb overlapping region was identified. In the overlapping region, there were 56 annotated genes. However, no genes were found to meet the criteria described in the “Materials and methods” section.

### Construction of regulatory networks for flavonoid biosynthesis

Flavonoids are secondary metabolites that are enriched in purple-fleshed sweet potato, with essential roles in plant growth and human health^2,39^. The flavonoid biosynthetic pathway is conserved in plants and has been extensively studied^40^. In this study, a method combining information from prior knowledge of biosynthetic pathway, eQTL mapping, and coexpression analysis was used to construct the regulatory network for flavonoid biosynthesis in the storage roots of sweet potato.

**Fig. 3:**
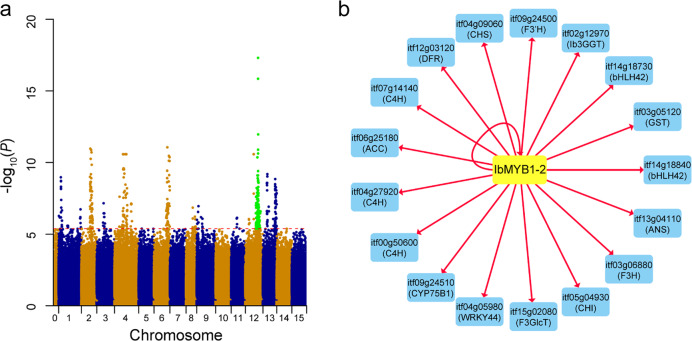
Regulatory network for the flavonoid biosynthesis pathway. **a** Combined Manhattan plot of SNPs associated with the expression of 39 genes involved in flavonoid biosynthesis. Green dots indicate that these loci were also significantly associated with the accumulation of anthocyanins in the storage roots of sweet potato. The red horizontal dashed line represents the genome-wide suggestive threshold (−log_10_(*P*) = 5.39, *α* = 1). **b** *IbMYB1-2* positively regulates 17 downstream genes in the flavonoid biosynthesis pathway. The yellow node indicates the candidate regulator, and blue nodes indicate regulated genes

Homology searches and subsequent manual checks of *I. trifida* gene models revealed 172 genes potentially involved in flavonoid biosynthesis (Supplementary Table S9, Supplementary Note). Based on the transcriptome data, 148 genes were found to be expressed. Furthermore, eQTL analysis showed that 39 genes had at least one significant eQTL (−log_10_ (*P*) = 5.39, *α* = 1). Among the genes with eQTLs, 11 genes were predicted to be regulated by only local eQTLs, 26 genes were regulated by only distant eQTLs, and 2 genes were regulated by both local and distant eQTLs. The comparison of intervals of the above eQTLs resulted in a set of 18 nonoverlapping regions (Fig. 3a). Interestingly, one of these regions was coincident with the QTL underlying the accumulation of anthocyanins in the storage roots of sweet potato (see the next section). Given the above evidence, we assumed that these QTLs are involved in flavonoid biosynthesis and contributed to the variation in flavonoid content in the storage roots of sweet potato.

By analyzing the genomic distribution of the above eQTLs, we found that several eQTLs for flavonoid biosynthesis-related genes were located in the same genomic region, suggesting that these eQTLs may contain the same regulatory factor controlling a suite of flavonoid biosynthesis-related genes. A method combining iterative group analysis (iGA)^41^ and coexpression analysis was used to identify regulatory groups and to construct a gene regulatory network for flavonoid biosynthesis. Only one significant regulatory group was identified (*P* = 2.29 × 10^−11^) (Table 1), and the regulatory relationships between regulator and target genes are shown in Fig. 3b. *IbMYB1-2*, which is known to regulate anthocyanin biosynthesis in tuberous roots^37,38^, was identified as a regulator controlling the expression of 17 target genes in our study. It was found that the target genes encode enzymes and transcription factors, such as *Chalcone isomerase* (*CHI*, itf05g04930), *Chalcone synthase* (*CHS*, itf04g09060), *Glutathione S-transferase* (*GST*, itf03g05120), *Anthocyanidin 3-O-glucoside-2*′*-O-glucosyltransferase* (*3GGT*, itf02g12970), and *Transparent testa 8* (*TT8*, itf14g18730). Some of these target genes, such as *3GGT* (named *Ib3GGT* in sweet potato)^42^, were reported to be directly activated by *IbMYB1-2*, which confirmed our results.

**Table 1:** Significant regulatory group identified in the analysis

Regulator	Regulator gene name	*P*-value changed^a^	Targets	PCC^b^	Target gene name
IbMYB1-2	*MYB-type transcription factor* (*MYB113*)	2.29 × 10^−11^	itf14g18840	0.95	*bHLH transcription factor 42* (*bHLH42*, *TT8*)
			itf03g05120	0.94	*Glutathione S-transferase 26* (*GST26*, *TT19*)
			itf13g04110	0.93	*Anthocyanidin synthase* (*ANS*, *TT18*)
			itf14g18730	0.92	*bHLH transcription factor 42* (*bHLH42*, *TT8*)
			itf03g06880	0.91	*Flavanone 3-hydroxylase* (*F3H*)
			itf02g12970	0.9	*UDP-glucose: flavonoid 3-o-glucosyltransferase* (*UF3GT*)
			itf05g04930	0.89	*Chalcone isomerase* (*CHI*, *TT5*)
			itf09g24500	0.89	*Cytochrome P450 75B1* (*CYP75B1*, *TT7*)
			itf15g02080	0.89	*UDP-glucosyl transferase 78D2* (*UGT78D2*)
			itf04g09060	0.87	*Chalcone synthase* (*CHS*, *TT4*)
			itf04g05980	0.86	*Transparent testa glabra 2* (*TTG2*, *WRKY44*)
			itf12g03120	0.83	*Dihydroflavonol 4-reductase* (*DFR*)
			itf09g24510	0.78	*Cytochrome P450 75B1* (*CYP75B1*, *TT7*)
			itf07g14140	0.76	*Cinnamate-4-hydroxylase* (*C4H*)
			itf00g50600	0.74	*Cinnamate-4-hydroxylase* (*C4H*)
			itf06g25180	0.61	*Acetyl-CoA carboxylase 1* (*ACC1*)
			itf04g27920	0.53	*Cinnamate-4-hydroxylase* (*C4H*)

^a^The *P*-value threshold was 0.05/(total number of putative regulators) = 2.74 × 10^−5^, where the total number of putative regulators was 1823

^b^Pearson’s correlation coefficient (PCC) between the expression values of gene pairs

The regulator and target genes obtained above were further used as bait genes to identify additional flavonoid biosynthesis-related genes. The expression analysis showed that 63 genes were significantly coexpressed with at least 17 bait genes (*P* < 0.01) (Supplementary Table S10). Among these genes, eleven genes were found to be involved in the flavonoid pathway, such as *Glabra 2* (*GL2*, itf06g17460)^43^, *Chalcone isomerase-like* (*CHIL*, itf12g23980)^44^ and *Production of flavonol glycosides 3* (*PFG3*, itf09g00480)^45^. However, most of them (52, 83%) had no reported function in flavonoid biosynthesis. Of the 63 genes, eQTLs for 22 genes were colocated with *IbMYB1-2*. On the basis of the above evidence, we assumed that these genes are flavonoid-associated genes; further experiments should be performed to confirm the regulatory relationships identified in our study.

### Association study of storage root flesh color in sweet potato

**Fig. 4:**
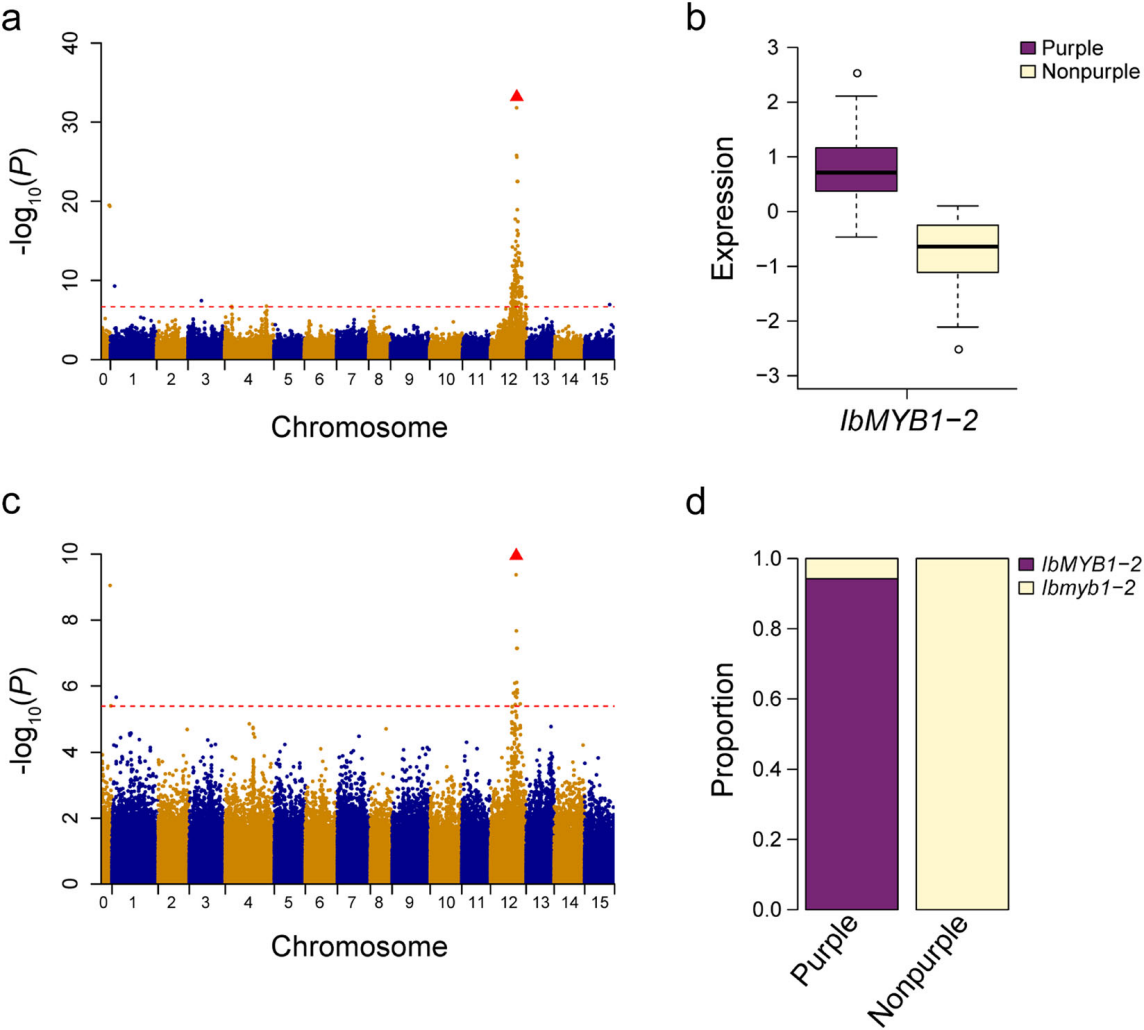
Genome-wide association for storage root flesh color in sweet potato. **a** Manhattan plot for storage root flesh color. The red horizontal dashed line represents the genome-wide significance threshold (−log_10_(*P*) = 6.68, *α* = 0.05). The red triangle corresponds to the proposed functional site (presence/absence variation of *IbMYB1-2*). **b** *IbMYB1-2* was significantly (*P* < 2.2 × 10^−16^) differentially expressed between purple and nonpurple sweet potato accessions. **c** Manhattan plot from the eQTL mapping of *IbMYB1-2*. The red horizontal dashed line represents the genome-wide suggestive threshold (−log_10_(*P*) = 5.39, *α* = 1). **d** Summary of the distribution of the *IbMYB1-2* mutation in sweet potato germplasm

Compared to traditional linkage mapping, association mapping is a more powerful approach for identifying causative variants responsible for phenotypic changes, especially in polyploid species^46^. In this study, a total of 104 sweet potato accessions, including 88 transcriptome sequencing accessions and 16 resequencing accessions, were used for GWAS. Considering the population structure and genetic relatedness among accessions, EMMAX^36^ was employed to perform genome-wide association for storage root flesh color. Our results showed that only one locus was significantly associated with flesh color at the genome-wide significance threshold (−log_10_ (*P*) = 6.68, *α* = 0.05) (Fig. 4a). This locus was located in the interval between 17,809,090 and 23,930,961 bp on chromosome 12, whose lead SNP was located at 20,657,886 bp with −log_10_ (*P*) = 31.82. Interestingly, the physical position of this locus (chr12: 17,809,090–23,930,961) was coincident with two types of eQTLs: one was a distant eQTL hotspot, and the other was an eQTL for flavonoid-related genes. Our eQTL hotspot and regulatory network analyses revealed that *IbMYB1-2* was the regulator controlling a suite of flavonoid-related genes. Furthermore, the expression levels of *IbMYB1-2* in purple-fleshed sweet potato accessions were significantly higher than those in nonpurple-fleshed sweet potato accessions (*P* < 2.2 × 10^−16^) **(**Fig. 4b). Based on these results, *IbMYB1-2* was the most promising candidate gene responsible for flesh color variation in sweet potato.

eQTL mapping detected one local eQTL for *IbMYB1-2* (Fig. 4c), indicating that sequence variation may be present in *cis*-regulatory elements of *IbMYB1-2*, such as the promoter, enhancer, or splice site. A previous study showed that there are three variants of *IbMYB1* (*IbMYB1-1*, *IbMYB1-2a*, and *IbMYB1-2b*) in sweet potato, and the presence/absence variation (PAV) of *IbMYB1-2a/b* was responsible for flesh color variation in the purple-fleshed cultivar ‘Ayamurasaki’ (presence of *IbMYB1-2a/b*, hereafter referred to as *IbMYB1-2*) and its spontaneous white-fleshed mutant ‘AYM96’ (absence of *IbMYB1-2a/b*, hereafter referred to as *Ibmyb1-2*)^38^. Using a pair of primers specific to the promoter of *IbMYB1-2* from cultivar ‘Ayamurasaki’^38^, we developed markers (Supplementary Table S2) to detect the PAV of *IbMYB1-2* in the GWAS population (Supplementary Table S1). The presence/absence polymorphisms of *IbMYB1-2* in the population were then recoded and integrated into the SNP genotype dataset. eQTL mapping revealed that the PAV of *IbMYB1-2* was the most significant locus (−log_10_ (*P*) = 9.95) associated with the expression levels of *IbMYB1-2*. The GWAS for flesh color showed that the PAV of *IbMYB1-2* was also the most significant signal (−log_10_ (*P*) = 33.18), and it explained 75.65% of the phenotypic variation. To further validate the most significant locus identified, the markers were used to screen a germplasm collection of 225 sweet potato accessions (94 purple-fleshed accessions and 131 non-purple-fleshed accessions, Supplementary Table S1). The results revealed that *IbMYB1-2* was strongly associated with flesh color (*P* = 2.06 × 10^−53^, Fisher’s exact test). However, eight purple-fleshed accessions were found to harbor *Ibmyb1-2*. The flesh color of these accessions was found to be either purple with white/orange rings and spots or orange/yellow with purple rings and spots, which was different from that of the other purple-fleshed accessions with anthocyanins covering all flesh, indicating that other mechanisms may contribute to this type of anthocyanin accumulation in storage roots. Overall, we concluded that *IbMYB1-2* is the major gene related to the activation of anthocyanin biosynthesis in the storage roots of sweet potato.

## Discussion

### SNP detection and expression profiling in hexaploid sweet potato

NGS-based SNP detection accelerates the development of genomic breeding strategies, such as genomic selection and genome-wide association studies^47,48^. Compared with that in diploid plants, the presence of polymorphisms among the highly similar subgenomes in polyploid plants makes accurate detection of SNPs more challenging. Despite the above difficulty, SNPs have been successfully used in some autopolyploid and allopolyploid plants, such as peanut^49^ and potato^50^. A complete genome for sweet potato is not yet available due to its high degree of heterozygosity and high polyploid level. In our study, the reference genome of the diploid progenitor *I. trifida* and a modified SWEEP algorithm^47^ were used to detect homologous SNPs across sweet potato accessions. The identified SNPs are of great importance to sweet potato research, especially using linkage and association mapping to uncover the genetic architecture of key agronomic traits in sweet potato.

Large-scale gene expression profiling data have been generated for several plant species among various tissue types, developmental stages, and stress treatments^13,15,19–21,23,51^. By analyzing RNA-seq data for mature storage roots of 88 sweet potato accessions, we found that gene expression profiles were highly variable. A large core set of genes (71.78% of all scored genes) were detected in all of the accessions, suggesting that these genes may play essential roles in the development of storage roots. However, almost one-fourth of the genes were found to be expressed in only a subset of the accessions, which are likely to be regulated by eQTLs. These gene expression profiles of sweet potato are a valuable resource for researchers that can be used to interpret gene functions and to understand storage root growth and development.

### Large-scale local and distant eQTLs are identified in storage roots

Gene expression variation is caused by *cis*- and/or *trans*-regulatory changes^52^. *Cis*-regulatory changes are mapped close to the regulated genes and are referred to as local eQTLs^53^. In contrast, *trans*-regulatory changes are mapped far from regulated genes and are referred to as distant eQTLs^53^. eQTL mapping at the genome-wide level has been conducted in many plants, such as Arabidopsis^19^, lettuce^20^, maize^13,21,22^, rice^15^, and tomato^23^. Some of these studies used relatively small population sizes (<100 individuals) to perform eQTL mapping but obtained meaningful results^18^. In this study, we performed eQTL mapping in 86 sweet potato accessions and detected 4408 eQTLs regulating the expression of 3646 genes. To the best of our knowledge, this is the first global eQTL analysis in sweet potato. Further results of our study showed that the identified eQTL was colocated with QTL associated with anthocyanin accumulation, suggesting that the 86 accessions included here were sufficient for identifying real association signals between SNPs and expression traits in sweet potato.

Our results revealed that eQTLs are not evenly distributed across the genome. By analyzing the distribution of distant eQTLs, 10 distant eQTL hotspots were found to regulate 158 target genes. eQTL hotspots may reflect regions that are either gene rich or infrequently recombined, which are generally of little functional interest^18^. Alternatively, eQTL hotspots may contain master regulators controlling the expression of functionally related genes^18^. In this study, two distant eQTL hotspots were found with target genes significantly overrepresented in certain biological processes. In the eQTL hotspot on chromosome 12, we found that *IbMYB1-2* functions as a master regulator and regulates the expression of 47 genes related to the phenylpropanoid metabolic process, flavonoid metabolic process, response to abiotic stimulus, etc. In tomato and lettuce, MYB transcription factors were reported to be master transcriptional regulators and major genes responsible for variation in flavonoid accumulation^20,23^. These results suggested a conserved regulatory role for MYB transcription factors in flavonoid biosynthesis. In the eQTL hotspot on chromosome 13, we did not find any potential master regulators. The comparative genomic analysis of cultivated sweet potato and *I. trifida* showed that only 88.92% of 10× Genomics linked-reads of *I. batatas* could be aligned to the *I. trifida* genome, indicating that genomic sequence variation is widespread between *I. trifida* and *I. batatas*^5^. We also found that *IbMYB1-2* was absent from the *I. trifida* genome (Supplementary Note). Based on the above evidence, we speculated that genome variation between *I. trifida* and *I. batatas* contributed to this result. Future eQTL analysis using the genome of *I. batatas* as the reference genome may identify the master regulator in this hotspot.

### eQTLs link genetic variation with phenotype in sweet potato

The QTLs identified by traditional linkage and association analysis can explain phenotype variation but are insufficient for revealing the underlying molecular mechanisms involved. eQTL studies, on the other hand, provide insights into how the genes of interest are regulated and help explore the relationship between phenotype and genotype^22^. The combination of eQTL and QTL analysis is an effective method for revealing the genetic architecture of complex traits in plants, such as flowering time^15^, anthocyanin content^20^, and maize kernel oil concentration^22^.

Anthocyanin content is an important quality trait in purple-fleshed sweet potato. Previous molecular and reverse genetic analyses have shown that *IbMYB1-2* participates in anthocyanin biosynthesis in the storage roots of sweet potato^37,38^. However, little is known about the genetic regulatory mechanisms of anthocyanin accumulation in the storage roots of sweet potato. As a representative example, we constructed a genetic regulatory network for flavonoid biosynthesis in sweet potato. Network analysis revealed many genes that may be related to flavonoid synthesis. Many of these genes are known to be related to flavonoid biosynthesis. There are also many genes with no reported function associated with flavonoid biosynthesis (Supplementary Table S10). In the future, studies on the function and mechanism of these genes will provide further insights into flavonoid biosynthesis and its regulation. The regulatory network and GWAS analysis were further combined to explore the genetic architecture of anthocyanin accumulation in storage roots. We found that one eQTL on chromosome 12 associated with flavonoid biosynthesis was colocalized with the QTL region identified by GWAS. We demonstrated that *IbMYB1-2* is the causative gene of anthocyanin biosynthesis activation in purple-fleshed accessions with anthocyanins covering all flesh. Future studies should focus on other types of purple-fleshed accessions to identify novel genes that can also induce the accumulation of anthocyanins in storage roots.

However, many eQTLs for the flavonoid biosynthesis genes were not included in the GWAS results. For example, *UDP-Glucosyltransferase 73B2* (*UGT73B2*, itf09g01890) has a local eQTL (on chromosome 9: 917,206–919,109). However, no signal was detected in this region using GWAS. This may suggest that glucosyltransferase is not the rate-limiting enzyme and that its regulation does not considerably change the flavonoid content. Alternatively, the expression variation may simply result in small changes in the final metabolites (anthocyanins). It would be interesting to study their molecular functions and investigate whether their expression differences affect anthocyanin accumulation in a well-controlled environment.

## Materials and methods

### NGS datasets

A total of 104 sweet potato accessions with anthocyanin variation in root flesh were used in this study (Supplementary Table S1). NGS data for these accessions were collected from two previous studies: one was a transcriptome dataset of mature storage roots of 88 sweet potato accessions^7^, and the other was a resequencing dataset of the Mwanga diversity panel containing 16 sweet potato accessions^5^. In our study, the transcriptome dataset was used only for eQTL mapping, and the transcriptome and resequencing datasets were combined for genome-wide association analysis of storage root flesh color. The raw data for these accessions were downloaded from the NGDC (https://bigd.big.ac.cn/)^25^ with accession number CRA000608 and the NCBI (https://www.ncbi.nlm.nih.gov/)^26^ with accession number SRP162006.

### Mapping and SNP genotyping

Raw fastq data were filtered to remove low-quality reads and sequencing adaptors using Trimmomatic (version 0.33)^54^. *I. trifida* (modified version, Supplementary Note)^5^, which is the closest diploid wild relative of sweet potato^4^, was used as the reference genome. For transcriptome data of 88 accessions and resequencing data of 16 accessions, the filtered fastq files were mapped to *I. trifida* using STAR (version 2.4.2a)^55^ and BWA-MEM (version 0.7.8)^56^, respectively. Then, PCR duplicates were removed using Picard tools (version 1.139).

The three steps described below were used to detect SNPs in hexaploid sweet potato. First, the mpileup command from SAMtools (version 1.2) was used to call raw SNPs^57^. Second, potentially false SNPs were filtered out using BCFtools according to our previous study^20^: (i) mapping quality, SNP quality and total read depth each had to be ≥30; (ii) any called SNP had to be located more than 3 bp away from InDels; (iii) any called SNP had to be biallelic; (iv) for homozygous SNPs, the read depth had to be ≥3, and the genotype quality had to be >20; (v) for heterozygous SNPs, the read depth for the reference and alternative allele had to be ≥2, and the genotype quality had to be >20; and (vi) SNPs that did not meet all of the above criteria were masked as missing genotypes. Third, the homeologous SNPs were filtered out by an in-house Perl script based on the following criteria: (i) SNPs had to have a missing rate ≤0.4; (ii) the genotypes at each SNP site had to be (0/0 and 0/1) or (0/1 and 1/1) in the population; (iii) each genotype had to be supported by at least five accessions; and (iv) sites that successfully passed the above criteria were used for further analysis.

### Assessing the functional impact of SNPs and GO enrichment analysis

SnpEff (version 4.1 l)^58^ was used to annotate and predict the effects of SNPs. Protein domain families were identified using the Pfam database^59^ at an e-value < 1e−5. Protein domain and GO enrichment analyses were performed using FuncAssociate (v3.0)^60^, agriGO (v2.0)^61^ and clusterProfiler^62^ with a cutoff of *P* < 0.05.

### Transcriptome analysis

Transcriptome analysis was performed for 88 sweet potato accessions. RSEM (version 1.3.0)^33^ was used to calculate the TPM value for each gene based on alignment to the *I. trifida* genes (high-confidence gene model set, version 3).

After TPM normalization, genes with nonzero TPM values in more than 50% of the accessions were retained to measure expression variability. The CV of each gene was then calculated. The highly variable genes were those that fell in the fourth quartile of the CV distribution.

### eQTL mapping

eQTL mapping was performed for 88 sweet potato accessions. PCA showed that 92.3% of the expression variance could be explained by the first three principal components (PCs). Two accessions (CRR022952 and CRR022965) that were greater than 2.5 standard deviations (SD) from the mean in any of the first three PCs were considered outlier samples and removed, resulting in a total of 86 accessions (*n* = 86) for eQTL mapping (Supplementary Table S1).

Genes were considered lowly expressed and thus were removed if they had a nonzero TPM value in <50% of samples and had a total TPM value less than 10. The qqnorm function implemented in R was applied to normalize the expression values of each gene^21,63^. SNPs with MAF ≥ 5% and missing rate ≤ 10% were used for eQTL mapping. The BN matrix was used to account for population structure and genetic relatedness among accessions. To further increase the power to detect eQTLs, six PEER factors that accounted for the variability of expression were treated as additional covariates^64^. Genome-wide eQTL mapping was performed using EMMAX software^36^. The effective number of independent SNPs (*n*) was calculated using GEC^65^. Suggestive *P*-value of 4.04 × 10^−6^ (*P* = *α*/*n*; where *α* = 1, *n* = 247,377) was used to identify significantly associated SNPs, with a corresponding −log_10_ (*P*) of 5.39. Manhattan plots were drawn using the R language.

To identify eQTL regions, the pairwise LD statistic *r*^*2*^ was calculated for the significant SNPs. Clusters with at least three significant SNPs were regarded as eQTL blocks if all pairwise *r*^*2*^ values between the SNPs were greater than 0.1. Otherwise, these SNPs were regarded as false positive signals. The SNP with the lowest association *P*-value in an eQTL block was designated as the lead SNP.

To classify the eQTLs as local eQTLs and distant eQTLs, the physical positions of the eQTLs and their target genes were compared. A local eQTL was identified if the eQTL spanned the target gene on the same chromosome. A distant eQTL was identified if the eQTL was located outside of the target gene on the same chromosome or on a different chromosome.

### Identification of distant eQTL hotspots

hot_scan software^66^ was used to identify significant distant eQTL hotspots, with the following parameters: -m 3000000 -s 0.05. Circos^67^ was used to display distant eQTL hotspots. The following criteria were developed to identify the candidate regulators in each eQTL hotspot: first, the candidate regulator had to reside in an eQTL hotspot; second, a local eQTL had to be detected for the candidate regulator; third, the PCC values between the candidate regulator and target genes had to be greater than 0.51 or less than −0.27 (*P* < 0.05); fourth, the candidate regulator in each distant eQTL hotspot had to be coexpressed with at least 50% of the target genes; and fifth, the candidate regulator had to be a transcription factor or signaling molecule.

### Regulatory network construction

Gene regulatory network was constructed according to our previous study^20^. The probability of change (PC) value for each gene located in the distant eQTL was calculated using the iGA algorithm^41^. The gene was classified as a candidate regulator if it had a Bonferroni-adjusted PC value less than 0.05/(total gene number in the eQTL region). Cytoscape^68^ was used to display the relationships between regulators and their target genes. The regulators and target genes were considered bait genes to identify additional genes associated with flavonoid biosynthesis. The pairwise PCC values between bait genes and all of the other expressed genes were calculated. Genes were considered to be coexpressed if their PCC value was greater than 0.53 or less than −0.49 (*P* < 0.01). A putative flavonoid biosynthesis-related gene was identified if it was coexpressed with at least 13 bait genes.

### Phenotyping and association analysis

Three replicates of all accessions (*n* = 225) were planted in an experimental field using a randomized complete block design in Xuzhou, Jiangsu Province, P.R. China, in 2019. Storage root flesh was classified as either purple or nonpurple (including white, cream, yellow or orange). For the 104 accessions, association analysis was conducted using EMMAX software^36^. Significant *P*-value of 2.08 × 10^−7^ (*P* = *α*/*n*; where *α* = 0.05, *n* = 240,466) was used to identify significantly associated SNPs, with a corresponding −log_10_ (*P*) of 6.68. For all 225 accessions, genomic DNA was extracted from young leaves using the CTAB method^69^. A pair of primers specific to the promoter of *IbMYB1-2* was used to screen the population. PCR was carried out in a 20 μL reaction containing 40 ng of genomic DNA as template, 0.5 μM of each primer, and 10 μL of 2 × Es Taq MasterMix (CWBIO). PCR was performed under the following conditions: denaturation at 94 °C for 2 min, followed by 35 cycles of amplification (94 °C for 20 s, 55 °C for 30 s and 72 °C for 30 s) and a final extension at 72 °C for 5 min.

## Supplementary information

Supplementary information

Supplementary tables
