# Supplementary material for: Genome-wide analysis of expression quantitative trait loci (eQTLs) reveals the regulatory architecture of gene expression variation in the storage roots of sweet potato
Source: Hortic Res. 2020 Jun 1;7:90. doi: 10.1038/s41438-020-0314-4 (PMC7261777; doi:10.1038/s41438-020-0314-4)
Supplement: Supplementary file 1 — Supplementary information [file 41438_2020_314_MOESM1_ESM.docx]

**Supplementary information for**

**Genome-wide analysis of expression quantitative trait loci (eQTLs) reveals the regulatory architecture of gene expression variation in the storage roots of sweet potato**

Lei Zhang, Yicheng Yu, Tianye Shi, Meng Kou, Jian Sun, Tao Xu, Qiang Li, Shaoyuan Wu, Qinghe Cao, Wenqian Hou, Zongyun Li

**Material Pages**

**Supplementary Note 2-4**

**Supplementary Figures 5-11**

**Supplementary Tables 12-12**

###### Supplementary Note: Integrating *IbMYB1-2* into the *Ipomoea trifida* genome to form a modified reference genome

A set of 171 genes potentially involved in flavonoid biosynthesis were identified by homology search and manual check in *I. trifida* genome. However, *IbMYB1*, a known gene encoding an R2R3-MYB transcription factor that regulate anthocyanin biosynthesis in sweetpotato [1; 2], was not presented in gene set. To make sure whether the *IbMYB1* was absent from *I. trifida* genome, we aligned the genomic sequences of *IbMYB1* (Accession Number AB576765) to the *I. trifida* genome using BLASTN with default parameters. The BLASTN results showed that no subject has query coverage > 50% and identity > 90%. Via this analysis, we ensured that *IbMYB1* was absent from *I. trifida* genome.

A previous study have shown that there are three variants of *IbMYB1* (*IbMYB1-1*, *IbMYB1-2a* and *IbMYB1-2b*) in sweetpotato, and the presence of *IbMYB1-2a/b* (Accession Number AB576766 and AB576767) was responsible for accumulation of anthocyanins in storage roots of recent Japanese cultivars [2]. The sequences of *IbMYB1-2a* and *IbMYB1-2b* were identical, except for a SNP existed in the second intron [2]. Genetic analysis revealed that *IbMYB1-2a* and *IbMYB1-2b* were on the same chromosome [2]. So, we referred these two genes as *IbMYB1-2* in our study. It was also shown that the CDS of *IbMYB1-1* and *IbMYB1-2* were identical, but flanking sequences of *IbMYB1-1* and *IbMYB1-2* (upstream and downstream of the CDS) were largely different [2]. The study also identified an *IbMYB1-2null* sequence (Accession Number AB588639) that are highly homologous to the 5’ and 3’ flanking regions of *IbMYB1-2*, and suggested that *IbMYB1-2* was likely been generated by an insertion of an *IbMYB1-1 type* sequence into *IbMYB1-2null* [2]. It further revealed that a single “**GTAAGTAC**” sequence was present at the putative insertion site of *IbMYB1-2null*, which was identical to target site duplication (TSD) of *IbMYB1-2* [2].

In our study, the combination of alignment and linkage disequilibrium (LD)-based methods was used to determine genomic positions of *IbMYB1-2* relative to the *I. trifida* reference genome.

First, since *IbMYB1-2* was likely been generated by a fragment been inserted into the *IbMYB1-2null* sequence, we aligned the sequences of *IbMYB1-2null* (300 bp upstream/downstream of putative insertion site) to the *I. trifida* reference genome (**Supplementary Fig. S1a**). The best hit was located on chromosome 12 at 20,714,068 bp -20,713,474 bp with pairwise identity of 92%, which also found to contain the “**GTAAGTAC**” sequence at 20,713,773 bp - 20,713,780 bp (**Supplementary Fig. S1b**). We assumed that the position of *IbMYB1-2* relative to the *I. trifida* genome was most likely located in the interval between 20,713,773 bp and 20,713,780 bp on chromosome 12.

Second, a LD-based approach was performed to identify chromosome positions of the *I. trifida* genome that were most tightly linked with *IbMYB1-2*. The determined chromosome position was considered as the approximate genomic position of *IbMYB1-2* relative to the *I. trifida* genome. Using a pair of primers that specific to the *IbMYB1-2* [2], we developed markers (**Supplementary Table S2**) to detect the PAV of *IbMYB1-2* in 104 sweetpotato accessions (**Supplementary Table S1**). The presence/absence polymorphisms of *IbMYB1-2* were then recoded and integrated into the SNP genotype dataset. The LD (*r*^2^) between PAV of *IbMYB1-2* and all other SNP genotypes were calculated and the physical position of the SNP in the *I. trifida* genome with the highest LD was regarded as the approximate chromosome location of *IbMYB1-2* (**Supplementary Fig. S1c**). Our results showed that the SNP with the highest LD was located at 20,657,886 bp on chromosome 12 (**Supplementary Fig. S1d**).

The alignment method can give accurate genomic position of *IbMYB1-2*, however, LD-based method can predict approximate genomic position of *IbMYB1-2*. The physical locations reported by these two approaches were very close, and can confirm each other. In our study, the result based on the alignment was considered as the final location of *IbMYB1-2*. The assigned genomic position of *IbMYB1-2* relative to the *I. trifida* genome was chr12: 20,713,773-20,713,780. The genomic sequence of *IbMYB1-2* was integrated into the reference genome to form a modified genome version of *I. trifida* for further analysis*.*

References

[1] H. Mano, F. Ogasawara, K. Sato, H. Higo, and Y. Minobe, Isolation of a regulatory gene of anthocyanin biosynthesis in tuberous roots of purple-fleshed sweet potato. Plant physiology 143 (2007) 1252-68.

[2] M. Tanaka, R. Kurata, H. Nakayama, and M. Yoshinaga, Structural and functional characterization of IbMYB1 genes in recent Japanese purple-fleshed sweetpotato cultivars. Molecular Breeding 29 (2012) 565-574.

###### Supplementary Figures:


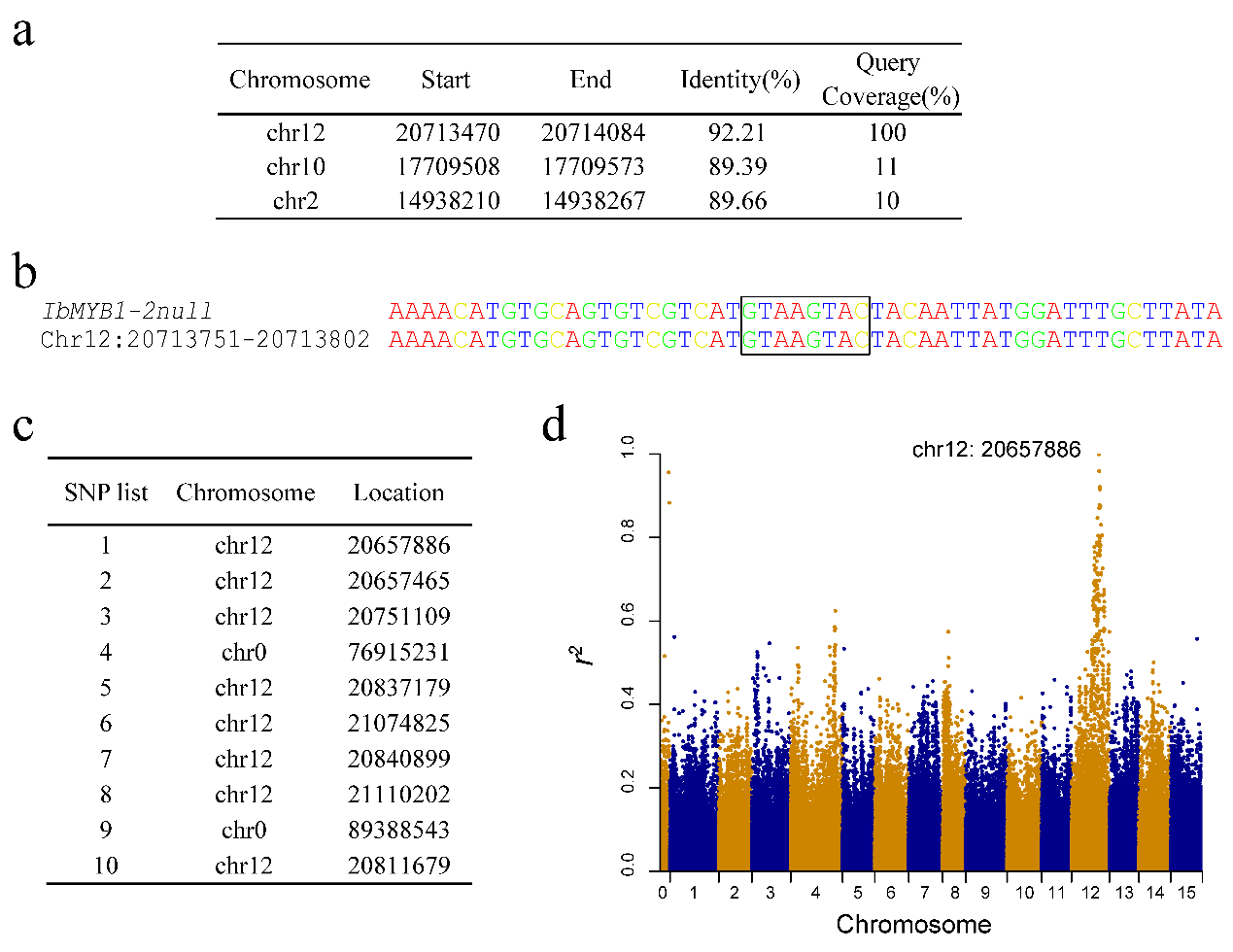


Supplementary Fig. S1: Determining genomic position of *IbMYB1-2* relative to the *I. trifida* reference genome

1. The top three blast hits for the *IbMYB1-2null* sequence when aligned to the *I. trifida* genome. (b) Sequence alignment of *IbMYB1-2null* and the best hit of *I. trifida*. The sequence in black box indicates the putative insertion site of “**GTAAGTAC**” sequence. (c) Chromosome locations most tightly linked with the *IbMYB1-2.* (d) The SNP with the highest LD has chromosome location of chr2: 20657886.


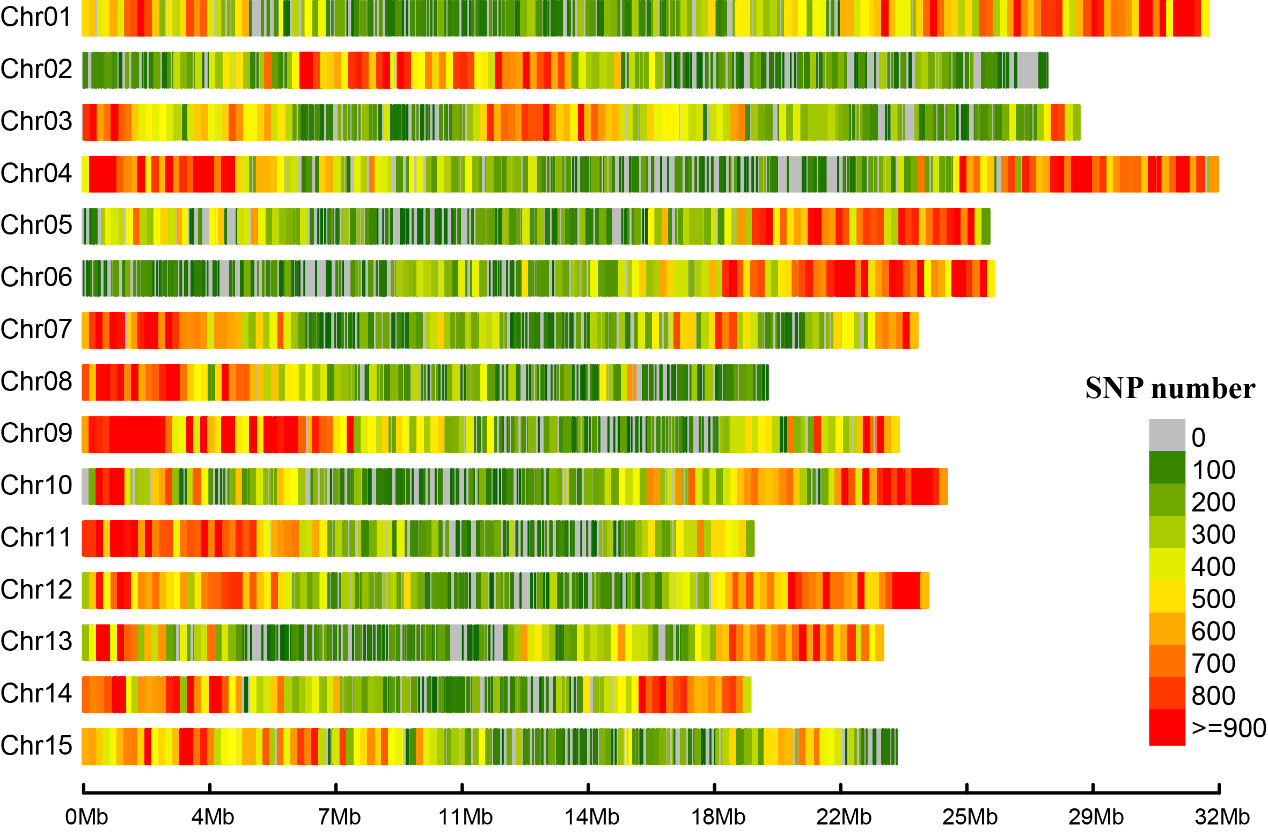


Supplementary Fig. S2: Distribution of SNPs in the sweetpotato genome

The distribution of SNPs was visualized across the sweetpotato chromosomes. The SNP density plots were performed using the R software package “CMplot” with the window size of 200 Kb.


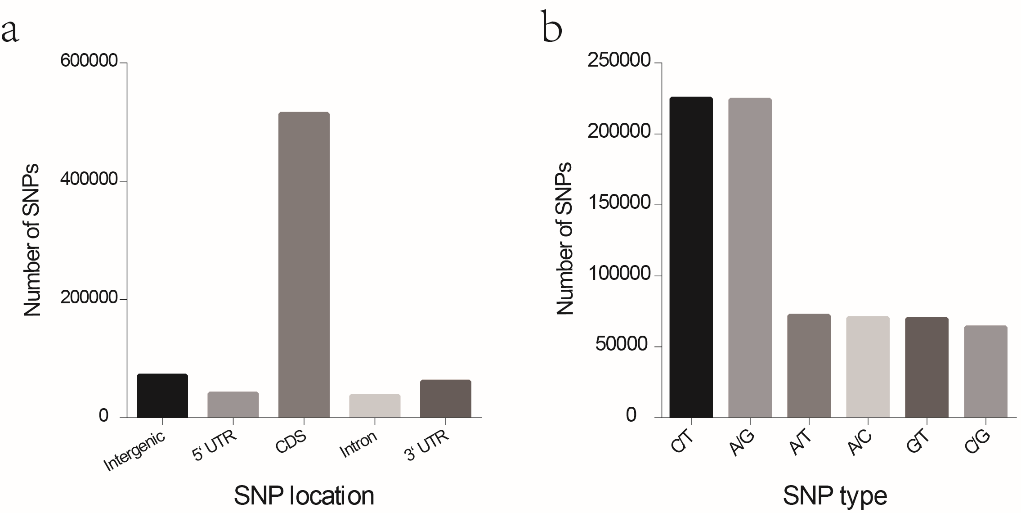


Supplementary Fig. S3: Characteristics of identified SNPs

(a) Number of SNPs located in each region. Upstream refers to a region that is within 3 Kb upstream of the start codon. Downstream refers to a region that is within 3 Kb downstream of the stop codon. (b) Number of each SNP type.


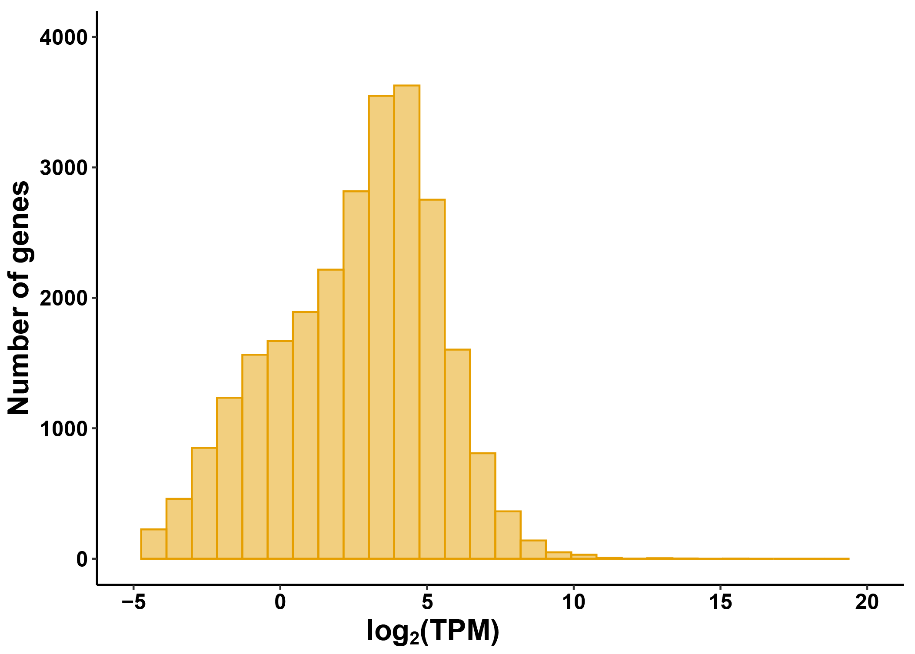


Supplementary Fig. S4：Histogram showing the distribution of gene expression values for all genes in 88 sweet potato accessions

*X*-axis is the log_2_-transformed mean TPM value per gene across all accessions. *Y*-axis indicates the number of genes.


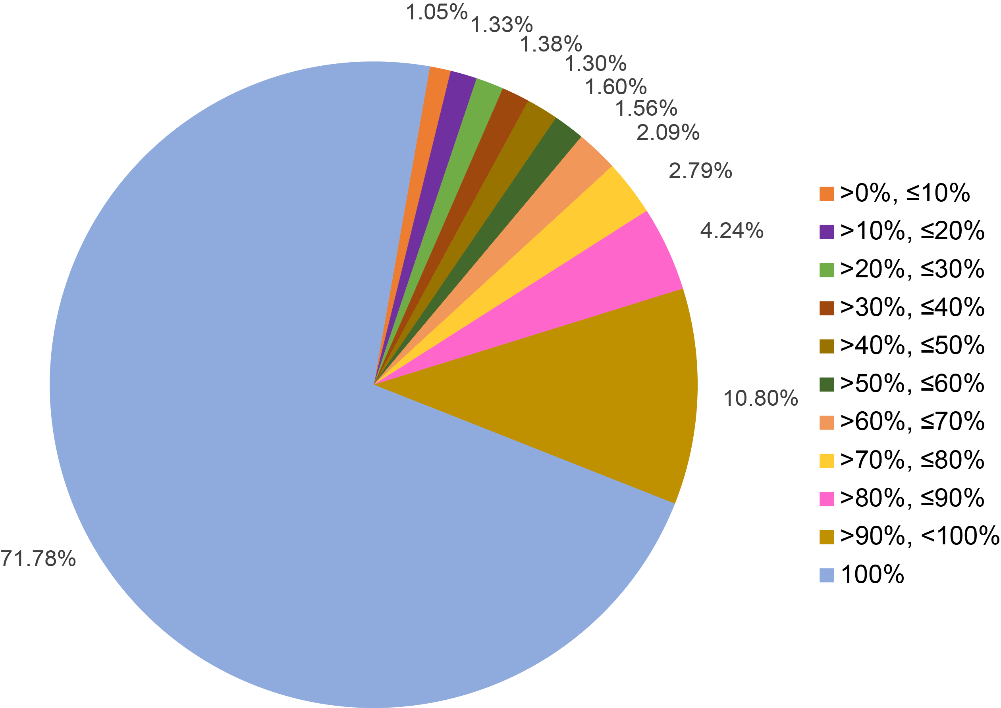


Supplementary Fig. S5：Percentage of genes detected in 88 sweetpotato accessions

A total of 18,681 (71.78%) expressed genes could be detected in all accessions.





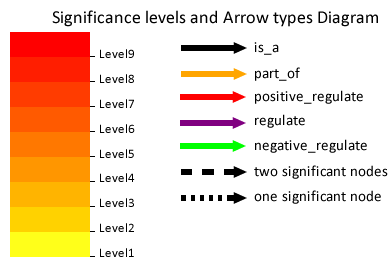


Supplementary Fig. S6：Directed acyclic graphs (DAGs) of GO enrichment analysis of highly variable genes on biological process


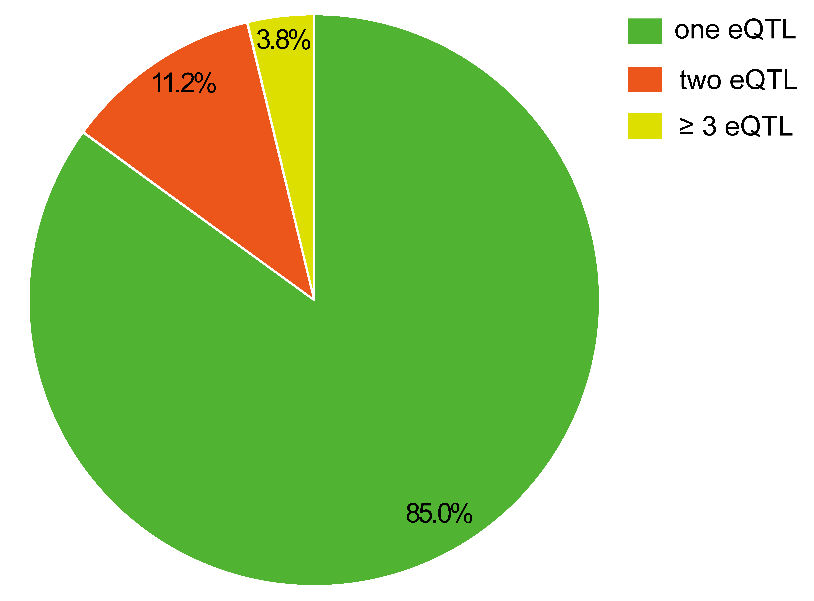


Supplementary Fig. S7: Percentage of genes with one or more eQTLs

###### Supplementary Tables:

Supplementary Table S1: Summary of the 225 sweetpotato accessions. (included as an excel file)

Supplementary Table S2: Primers used in this study. (included as an excel file)

**Supplementary Table S3:** List of SNPs that have a high impact on gene functions. (included as an excel file)

**Supplementary Table S4:** Pfam domain enrichment analysis of large-affected genes. (included as an excel file)

Supplementary Table S5: List of 100 most highly expressed genes in mature storage roots. (included as an excel file)

**Supplementary Table S6:** Pfam domain enrichment analysis of 100 most highly expressed genes in mature storage roots. (included as an excel file)

Supplementary Table S7: List of identified eQTL. (included as an excel file)

Supplementary Table S8: List of identified distant eQTL hotspots. (included as an excel file)

Supplementary Table S9: Genes involved in flavonoid biosynthesis in sweetpotato. (included as an excel file)

Supplementary Table S10: Other genes possibly associated with flavonoid biosynthesis. (included as an excel file)
